# Annual acknowledgement of manuscript reviewers 2014

**DOI:** 10.1186/s13722-015-0025-6

**Published:** 2015-05-01

**Authors:** Jeffrey Samet

**Affiliations:** Addiction Science & Clinical Practice, London, UK

## Abstract

The editor of *Addiction Science & Clinical Practice* would like to thank all reviewers who have contributed to the journal in Volume 9 (2014).

**Marc Auriacombe**

France

**Preben Bendtsen**

Sweden

**Nicolas Bertholet**

Switzerland

**Randall Brown**

United States of America

**Spencer Bujarski**

United States of America

**Debbie Cheng**

United States of America

**Jan Copeland**

Australia

**John Cunningham**

Australia

**Itai Danovitch**

United States of America

**Carol Dawson-Rose**

United States of America

**William DeJong**

United States of America

**Michelle Drapkin**

United States of America

**Quyen Epstein-Ngo**

United States of America

**Loretta Finnegan**

United States of America

**Niamh Fitzgerald**

United Kingdom

**Jacques Gaume**

Switzerland

**Stuart Gitlow**

United States of America

**Traci Green**

United States of America

**Kevin Haggerty**

United States of America

**Jan Kamphuis**

Netherlands

**Robert Kempainen**

United States of America

**Megan Lim**

Australia

**Jan Lindsay**

United States of America

**Karsten Lunze**

United States of America

**Jim McCambridge**

United Kingdom

**Anna McDani**

United States of America

**Jennifer Merrill**

United States of America

**Nicola Metrebian**

United Kingdom

**Jane Metrik**

United States of America

**Karen Miotto**

United States of America

**Willscott Naugler**

United States of America

**Josiah Rich**

United States of America

**Rutherfoord Rose**

United States of America

**Andrew Saxon**

United States of America

**GM Schippers**

Netherlands

**J.D. Smith**

United States of America

**Cindy Thomas**

United States of America

**Steven Zeliadt**

United States of America

